# Non-contrast CT synthesis using patch-based cycle-consistent generative adversarial network (Cycle-GAN) for radiomics and deep learning in the era of COVID-19

**DOI:** 10.1038/s41598-023-36712-1

**Published:** 2023-06-29

**Authors:** Reza Kalantar, Sumeet Hindocha, Benjamin Hunter, Bhupinder Sharma, Nasir Khan, Dow-Mu Koh, Merina Ahmed, Eric O. Aboagye, Richard W. Lee, Matthew D. Blackledge

**Affiliations:** 1 Division of Radiotherapy and Imaging, the Institute of Cancer, London, SM2 5NG UK; 2 AI for Healthcare Centre for Doctoral Training, Imperial College London, Exhibition Road, London, SW7 2BX UK; 3 Department of Radiology, The Royal Marsden NHS Foundation Trust, Sutton, SM2 5PT UK; 4 Cancer Imaging Centre, Department of Surgery & Cancer, Imperial College London, Du Cane Road, London, W12 0NN UK; 5 Early Diagnosis and Detection Team, The Royal Marsden NHS Foundation Trust, Fulham Road, London, SW3 6JJ UK; 6 Lung Unit, The Royal Marsden NHS Foundation Trust, Sutton, SM2 5PT UK

**Keywords:** Medical imaging, Medical research

## Abstract

Handcrafted and deep learning (DL) radiomics are popular techniques used to develop computed tomography (CT) imaging-based artificial intelligence models for COVID-19 research. However, contrast heterogeneity from real-world datasets may impair model performance. Contrast-homogenous datasets present a potential solution. We developed a 3D patch-based cycle-consistent generative adversarial network (cycle-GAN) to synthesize non-contrast images from contrast CTs, as a data homogenization tool. We used a multi-centre dataset of 2078 scans from 1,650 patients with COVID-19. Few studies have previously evaluated GAN-generated images with handcrafted radiomics, DL and human assessment tasks. We evaluated the performance of our cycle-GAN with these three approaches. In a modified Turing-test, human experts identified synthetic vs acquired images, with a false positive rate of 67% and Fleiss’ Kappa 0.06, attesting to the photorealism of the synthetic images. However, on testing performance of machine learning classifiers with radiomic features, performance decreased with use of synthetic images. Marked percentage difference was noted in feature values between pre- and post-GAN non-contrast images. With DL classification, deterioration in performance was observed with synthetic images. Our results show that whilst GANs can produce images sufficient to pass human assessment, caution is advised before GAN-synthesized images are used in medical imaging applications.

## Introduction

Since the COVID-19 pandemic, significant attention has been directed towards developing medical imaging-based Artificial Intelligence (AI) models for rapid diagnosis, risk-stratification and prediction of complications for this disease^1–5^. Computed tomography (CT) remains the imaging modality of choice for assessment of patients with moderate to severe features of COVID-19, for confirmed cases with worsening respiratory status, and for patients with functional impairment or hypoxia after recovery from COVID-19^6^.

Radiomics models, either using hand-crafted features^7^ or deep learning (DL) methods, may facilitate the development of advanced imaging analysis tools for diagnosis and prognostication in COVID-19 research^3,5,8^. Through the extraction of quantitative features from standard-of-care medical imaging, they offer non-invasive biomarkers that can guide clinical decision-making^9–11^. Such algorithms are reliant on large and high-quality annotated datasets^12,13^. However, heterogeneity is inherent in medical imaging datasets, for example due to variation in scanning protocols, contrast enhancement and acquisition parameters including slice thickness and reconstruction kernels between centres^14,15^. This represents a common challenge which can introduce confounding factors that may impair a model’s ability to learn effectively from the data and perform reliably^16–22^. Imaging biomarker standardization efforts are underway to mitigate against this^23–25^. Contrast-enhanced CT imaging is a key example. Its use is clinically dictated and correlates with other variables which are likely to influence model performance^26^. Furthermore, patient related factors including physiology can significantly impact upon the resultant images between patients injected with the same contrast dose under the same imaging protocol^26^. Simply excluding images based on contrast status introduces bias, however datasets with a combination of contrast and non-contrast CT images can lead to signal intensity distribution shifts in training data which may impair performance of CT imaging-based predictive models^27^. This can influence the development of clinically relevant computational algorithms.

Imaging guidance for COVID-19 advises single-phase CT with no contrast enhancement or post-contrast series, as radiological features of COVID-19 tend to be confined to the lungs, without involvement of the pleura or mediastinum^6^. Direct post-contrast arterial-phase CT is recommended in the context of suspected pulmonary embolism or superimposed bacterial pneumonia, which are the primary differential diagnoses and common complications of severe COVID-19 cases^6,28^. Patients with COVID-19 who meet indications for CT imaging are likely to be breathless, coughing or clinically unstable, placing importance on shorter scanning protocols^6^. If comparable quality non-contrast images could be synthesized from contrast-enhanced CT images, this could enable standardization of contrast and non-contrast imaging for radiomics and DL algorithm training and validation.

Generative adversarial networks (GANs) present a potential solution^29^. GANs have gained popularity in data synthesis due to their ability to learn global context from large training examples^30,31^. In medical imaging, they facilitate domain adaptation for AI-based networks^29,32^. Choi et al. developed a three-dimensional (3D) conditional GAN (cGAN or pix2pix) to generate synthetic non-contrast chest CTs, demonstrating higher lesion conspicuity for expert reviewers in mediastinal lymph node assessments when synthetic images were used alongside acquired contrast CTs^33^. However, whilst their method produced predictions with higher perceptual quality than previous non-adversarial convolutional neural network (CNN)-based networks^34^, it still relies on paired input images which may not be feasible for real-world applications. The cycle-consistent GAN (cycle-GAN)^35^ has emerged as a revolutionary training strategy for generating photo-realistic and high-resolution synthetic images using unpaired data^36–38^. Chandrashekar et al. deployed a two-dimensional (2D) cycle-GAN to synthesize contrast CT angiograms (CTAs) to negate contrast CTA acquisition^39^. Xie et al. developed a 3D residual cycle-GAN to predict contrast-enhanced CTs for anatomy localization as well as organ-at-risk (OAR) delineations for radiotherapy treatment planning, without the need to perform contrast CT imaging^40^. Conversely, Sandfort et al. developed a cycle-GAN model as a data augmentation tool and demonstrated that the addition of synthetic non-contrast CT images in multi-organ segmentation training improved performance for out-of-distribution patient scans^41^. This is especially pertinent when considering the challenges around using COVID-19 imaging data to develop accurate and generalizable AI models. Therefore, synthesizing non-contrast CT images from contrast enhanced CTs has both clinical and research applications, which merit further development in DL technology. Non-contrast CT images can also be generated using non-AI techniques, such as acquiring the images using dual-energy CT^42^. The paired images acquired using different energies can be used to generate a virtual subtraction non-contrast image^42^. However, access to dual energy or multiband CT scanners and radiologist expertise is costly and not widely available, and therefore the development of AI techniques is an important solution.

Whilst previous research demonstrates a promising outlook for the integration of image synthesis in clinical decision-making, in-depth investigation of the performance of these algorithms on large multi-centre and contrast heterogenous COVID-19 datasets remains unexplored^31^.

In this study, we sought to close this research gap with the following contributions. Firstly, we developed a 3D patch-based cycle-GAN to synthesize non-contrast images from contrast CTs. We used 2,078 scans from 1650 patients with COVID-19 pneumonia, and qualitatively evaluated our results with a modified Turing-test for blind classification of synthetic and acquired CT images. We subsequently performed both a handcrafted radiomics and VGG-Net^43^ DL classification task to assess network generalizability as a data homogenization tool. We believe that our study on a large scale, multi-centre COVID-19 dataset provides a valuable evaluation of cycle-GAN generated synthetic CT data in the context of wider AI applications.

## Methods

### Datasets and ethical approval

**Figure 1:**
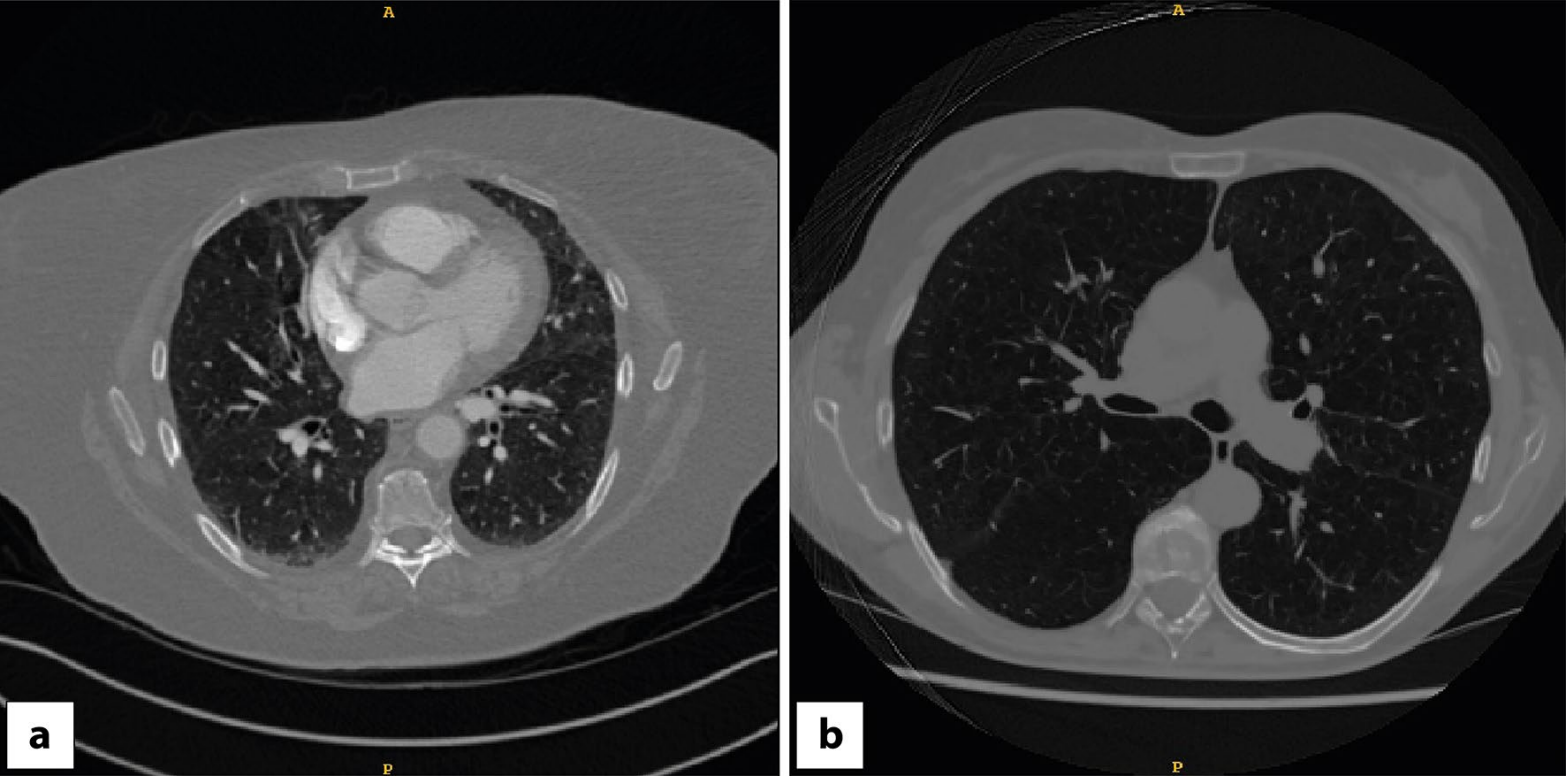
Examples of (**a**) contrast-enhanced and (**b**) non-contrast CTs from the NCCID.

This study was approved by the UK Health Research Authority (HRA) (reference number: 20/HRA/3051), ClinicalTrials.gov identifier: NCT04721444. CT images used for this study were obtained from the National COVID-19 Chest Imaging Database (NCCID)^44^ (Research Ethics Council (REC) reference number: 20/LO/0688). Patient consent was not required to use this deidentified data as per the respective UK Health Research Authority and UK Research Ethics Council approvals. All methods were performed in accordance with the relevant guidelines and regulations. Informed consent was obtained from the human experts involved in the study. NCCID is a centralized database containing medical images of hospital patients from over 25 centres across the UK. Images were filtered to include only CT images that included the entire thorax. For example, CT coronary angiograms were excluded. 2,078 CT scans were downloaded together with available metadata and manually labelled as either contrast-enhanced or non-contrast by a clinician (Fig. 1).

### Cycle-GAN framework

This section details how the 2078 labelled NCCID CT images were pre-processed and describes the development of the cycle-GAN framework including architecture, training, and inference.

#### Image pre-processing

From the 2,078 downloaded CT scans, low-dose CT scans, which constituted less than 3% of the dataset were excluded. The remaining 2,019 CT images (from 1650 patients) were resampled to $1.0\times 1.0\times 2.0{ \mathrm{mm}}^{3}$ voxel spacing using bilinear interpolation^45^. Intensity values outside the range of −1,000 to 1,000 Hounsfield units (HU) were truncated, and the remaining values were scaled to (−1.0, 1.0) following division by 1,000 to shorten the dynamic range for cycle-GAN training.

#### Network architecture

Conventionally, GANs consists of a generator and a discriminator, whereby the generator predicts synthetic images from random noise for the discriminator to classify as synthetic or real, during training. In a cycle-GAN^35^, images from domain A are synthesized to the domain B distribution and then reconstructed back to domain A to maintain spatial consistency. This process is simultaneously conducted for domain B synthesis and reconstruction, requiring two generators and two discriminators. Concurrent training of the generator and discriminator generates sets of trained weights that yield realistic synthetic images, which are indistinguishable from real images by the discriminator. Thereby, cycle-GAN promises to be an ideal strategy for style transfer from contrast to non-contrast CT, (and vice versa), using unpaired training data. This is particularly appealing for large multi-centre datasets that constitute large heterogeneity in CT images.

**Figure 2:**
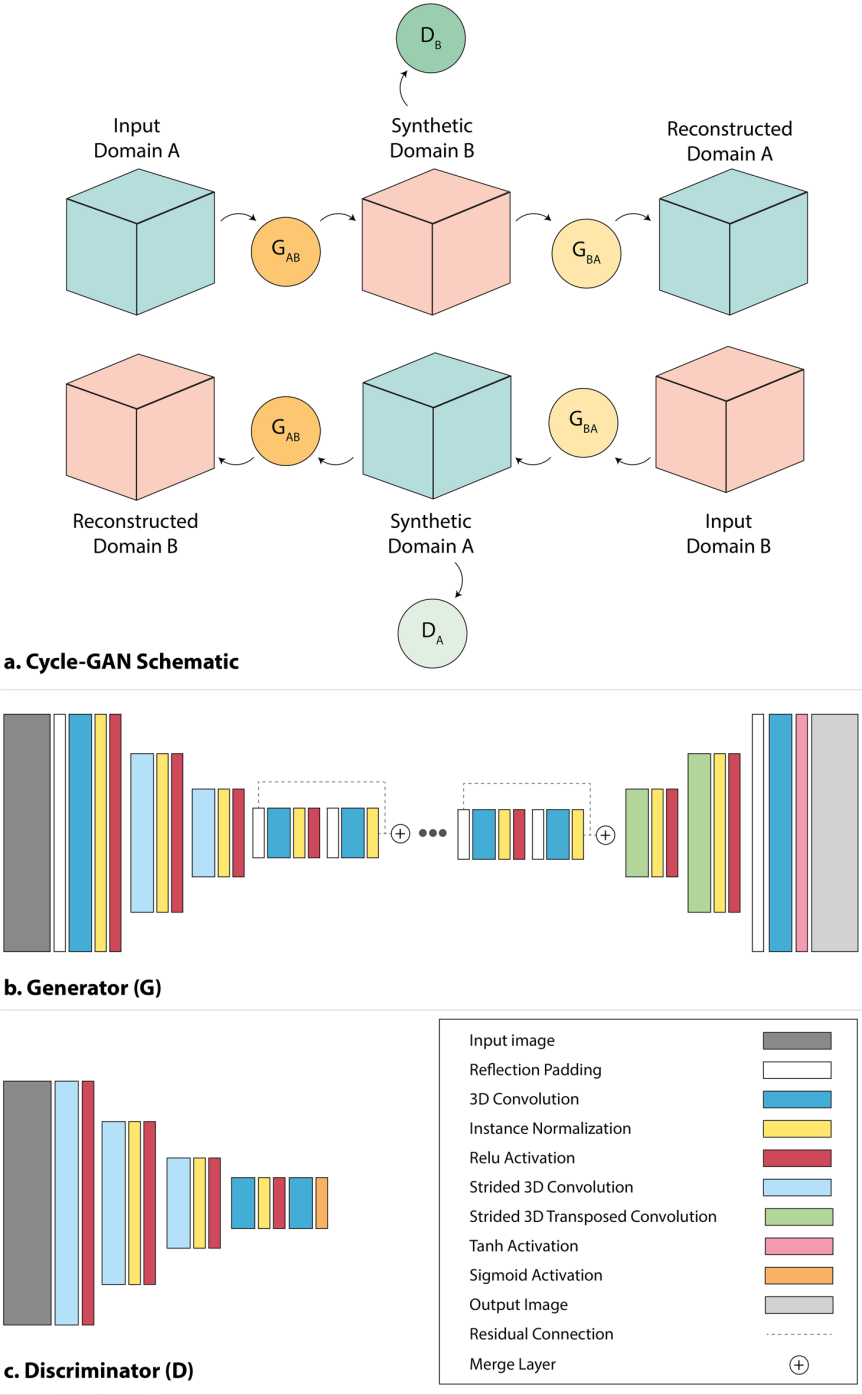
Cycle-GAN architecture—(**a**) Cycle-GAN schematic: image patches from domain A and domain B are respectively fed into the GAB and GBA generators to predict synthetic images. Subsequently, synthetic images are (1) reconstructed into their respective original domain, and (2) classified as synthetic or real by the DA and DB discriminators. Convergence is achieved when the discriminators are unable to distinguish between synthetic and real input CTs. For visual clarity, the operational blocks in the (**b**) generator and (c) discriminator architectures are presented as non-cubic blocks.

We developed a 3D patch-based cycle-GAN where the convolutional operations in the generators (G_AB_ and G_BA_) and discriminators (D_A_ and D_B_) were performed using 3D layers (Fig. 2). Our generator architecture was inspired by the Res-Net model in the vanilla cycle-GAN paper^35^ and consisted of one convolutional encoding block (ReflectionPad-Conv3D-InstanceNorm-Relu), two down-convolution blocks (StridedConv3D-InstanceNorm-Relu), nine residual units, two up-convolutional blocks (StridedTransposedConv3D-InstanceNorm-Relu), and a final Tanh activation layer. The patch-GAN discriminator included three down-convolutional blocks (StridedConv3D-InstanceNorm-LeakyRelu), one 3D convolutional layer and a final sigmoid activation layer. The computational graph for the models and the training code were implemented using TensorFlow 2.4.1 and Keras libraries.

#### Loss functions

The adversarial loss is an essential component of the cycle-GAN loss function that uses the minimax loss between real and synthesized images in domains A and B^29^ (Eqs. 1–3). To maintain spatial consistency in cycle-GAN predictions, cycle consistency loss was used. This term was defined as the average of mean absolute error (L_1_) and the structural similarity index (SSIM) loss^46^ between the input and reconstructed patches (Eq. 4). SSIM is an established and differentiable metric for evaluating image quality^46^. Additionally, a weighted identity loss term ($\lambda =10$) was included in the overall cycle-GAN loss to enforce sensitivity to both domains (Eq. 5). The overall cycle-GAN generator loss is presented in Eq. (6). During training, the patch-GAN discriminator loss was the mean squared error (L_2_) for classifying real and synthetic patches.

1 $${\mathcal{L}}_{a\mathrm{dversarial}(\mathrm{A}) }= {E}_{a\sim p(a)}\left[\mathrm{log}{D}_{A}(a)\right]+{E}_{b\sim p(b)}\left[\mathrm{log}(1-{D}_{A}({G}_{BA}\left(b\right))\right]$$

2 $${\mathcal{L}}_{\mathrm{adversarial}(\mathrm{B}) }= {\mathrm{E}}_{\mathrm{b}\sim \mathrm{p}(\mathrm{b})}\left[{\mathrm{logD}}_{\mathrm{B}}(\mathrm{b})\right]+{\mathrm{E}}_{\mathrm{a}\sim \mathrm{p}(\mathrm{a})}\left[\mathrm{log}(1-{\mathrm{D}}_{\mathrm{B}}({\mathrm{G}}_{\mathrm{AB}}\left(\mathrm{a}\right))\right]$$

3 $${\mathcal{L}}_{a\mathrm{dversarial}(\mathrm{A},\mathrm{B}) }= {\mathcal{L}}_{a\mathrm{dversarial}(\mathrm{A}) }+ {\mathcal{L}}_{a\mathrm{dversarial}(\mathrm{B})}$$

4 $${\mathcal{L}}_{cycle-consistency}= ({\mathcal{L}}_{1} +{\mathcal{L}}_{SSIM})/2$$

5 $${\mathcal{L}}_{identity}= {E}_{a\sim p(a)}\left[||{G}_{BA}\left(a\right)-a||\right]+{E}_{b\sim p(b)}\left[||{G}_{AB}\left(b\right)-b||\right]$$

6 $${\mathcal{L}}_{overall cycle-GAN loss}= {\mathcal{L}}_{adversarial(A,B)} {+ {\mathcal{L}}_{cycle-consistency}+ \lambda \times \mathcal{L}}_{identity}$$

where a and b represent domains A and B, G_AB_ and G_BA_ are the generators, and D_A_ and D_B_ denote the discriminators.

#### Training

From the 2,019 CT images (1,650 patients) in the NCCID dataset, 1,171 contrast CTs (929 patients) and 607 non-contrast CTs (522 patients) were used for training, and 119 contrast CTs (100 patients) and 126 non-contrast CTs (100 patients) for testing. During cycle-GAN training, random patches of size $64\times 64\times 64$ were selected from the resampled training image volumes. The network training was performed on an NVIDIA RTX6000 GPU (Santa Clara, California, USA) for approximately 450,000 iterations (batch size = 1; G&D Adam optimizer, learning rate = 2 × 10^–4^). Network training parameters were informed by our previous work^38^.

#### Inference

**Figure 3:**
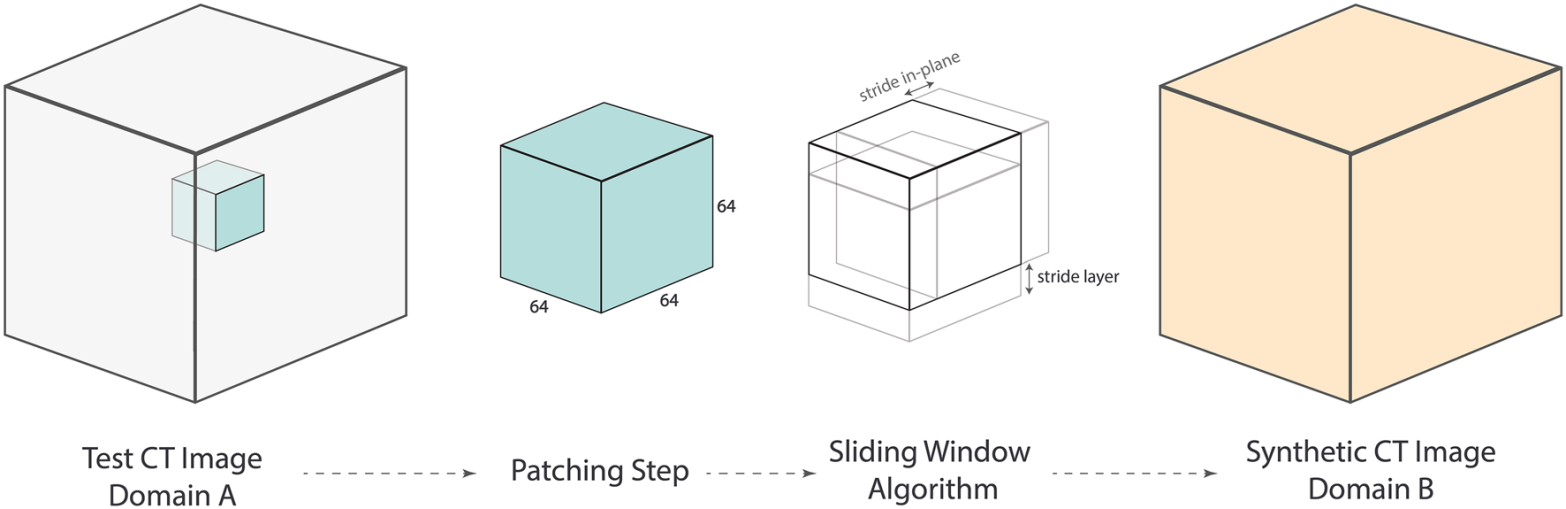
The sliding window inference algorithm used to predict synthetic patches by moving across the entire CT image volume with pre-determined strides.

During the evaluation phase, synthetic CT volumes were generated using a sliding window algorithm with a stride of 16 voxels (Fig. 3). Intensity averaging for overlapping regions in adjacent patches was applied so that the predicted voxels in the middle of each patch carried larger weighting than those from the borders. First, the inference algorithm was performed on acquired contrast CT scans to generate synthetic non-contrast images. Then, the non-contrast to contrast generator was used to regenerate contrast CTs. The absolute intensity difference maps between the normalized contrast/synthetic non-contrast and contrast/synthetic contrast CTs were generated for improved visualization of contrast removal from the test images (range: −0.4, 0.4).

#### Expert human reader assessment

To assess the photorealism of the synthetic non-contrast images, we undertook a modified Turing-test. Two radiologists and a clinical oncologist, with 43 years of cumulative experience since Fellowship of the Royal College of Radiologists (FRCR) accreditation were asked to review and classify 200 non-contrast single-slice thoracic CT images. 100 images were acquired CT slices and 100 were synthetic non-contrast CT slices generated from our cycle-GAN model, windowed to −1200 and 200 HU. The individual scores were recorded, and percentages and Fleiss’ Kappa^47^ were calculated to determine classification accuracy, sensitivity, specificity, and interrater agreement reliability respectively (Eqs. 7–9).

7 $$k= \frac{\overline{P}-{\overline{P}}_{e}}{1-{\overline{P}}_{e}}$$

where:

8 $$\overline{P}=\frac{1}{Nn(n-1)}\sum_{i=1}^{N}\sum_{j=1}^{k}{n}_{ij}({n}_{ij}-1)$$

9 $${\overline{P}}_{e}=\sum_{j=1}^{k}{\left(\frac{1}{Nn}\sum_{i=1}^{N}{n}_{ij}\right)}^{2}$$

where N is the total number of image slices, n is the number of expert readers and k represents the number of categories (synthetic vs acquired).

#### Validation with a VGG16 feature classifier

In addition to the human reader study, we developed an automated binary classifier for discriminating synthetic non-contrast from acquired non-contrast CT scans using a transfer learning paradigm. The classifier consisted of a fully connected network with node sizes of 256, 128, 64, 32, and 16 for consecutive hidden layers (sigmoid activation, ‘He’ uniform weight/bias initialization, and L_1_ weight/bias regularization applied on all nodes), and a final sigmoid layer for classification. As input to the classifier, we extracted features from each image slice using the pre-trained VGG16 model^48^; after removing the original classification layer of the VGG16 network and max-pooling across each channel in the preceding layer this resulted in 512 extracted features. To ensure that the features represented regions within the body only, an automatic body contour algorithm was applied prior to feature extraction, consisting of image thresholding (HU > −500), followed by morphological opening ($5\times 5$ square kernel) and hole filling. Images were subsequently normalized to the range $y\in [0, 1, ..., 255]$ using Eq. (10): $y = \left\lfloor {255 \times \left( {x + 1000} \right) / 2000} \right\rfloor$, where $x$ represents the CT number, and the result was replicated across all red, green, blue channels required for input to the VGG16 network (values greater than 255 or less than 0 were clipped).

Using a single slice from each of 200 test patients held back from the NCCID dataset in cycle-GAN training (100 contrast and 100 non-contrast), we trained three classifiers: (i) acquired contrast versus acquired non-contrast, (ii) synthetic non-contrast versus acquired non-contrast, and (iii) acquired contrast versus acquired non-contrast where labels were shuffled to derive performance statistics for a random choice model (i.e. to generate a Null hypothesis). These 200 patients were split into training/test datasets using an 80:20 ratio by stratified sampling. Using the 160 patient training data, the classifier was trained for 2,500 epochs, using Adam optimization (learning rate 1 × 10^–4^), a binary cross entropy loss function, batch size of 28, and validation data size of 48 patients (split evenly between both classes). To determine the distribution of training/validation curves, each of the models were trained in this way 30 times, in each instance randomly sampling 48 different validation patients. The mean and standard error in the mean of the training/validation curves at each epoch were recorded.

#### Validation with a handcrafted radiomics classifier

We undertook an additional assessment by comparing AUC values of machine learning classifiers trained using handcrafted radiomic features to predict COVID-19 vs non-COVID-19 pneumonia from single-slice CT images. Twin datasets were produced for this experiment:

- A “contrast-heterogenous” dataset—this combined CT images from 100 COVID-19 patients held back from the NCCID dataset in cycle-GAN training (50 contrast and 50 non-contrast) and 100 patients with non-COVID-19 pneumonia (30 contrast and 70 non-contrast) (further information is detailed in the Supplementary Material).
- A “contrast-homogenous” dataset—the contrast-enhanced images were replaced by the GAN-generated synthetic non-contrast equivalents (50 COVID-19 and 30 non-COVID-19 patients). For consistency and to exclude potential artifacts from the generators, acquired CTs were replaced by their corresponding predictions from the contrast to non-contrast model. Note that the identity term in the cycle-GAN loss function for network training ensured that the non-contrast images remained unaffected.

The images were resampled to a $1.0\times 1.0\times 1.0 {\mathrm{mm}}^{3}$ resolution using bilinear interpolation^45^ and single slice lung regions of interest (ROIs) encompassing diseased parenchyma were manually segmented by a clinician using the ITKSnap desktop software^49^. The radiomic features were standardized and extracted using TexLAB 2.0^50^. This was achieved using 25 HU intensity bins and for features broadly related to volume, intensity, heterogeneity and wavelet transformations, as previously described^50^. The subjects were divided into training and validation sets with a 4:1 ratio (training: 160, validation: 40).

Starting with the heterogenous dataset, highly correlated features were removed (threshold: 0.9) leaving 122 features, and then feature selection was performed using Kendall’s rank (threshold: 0.2) identifying 24 features (detailed in supplementary material). Seven machine learning classifiers (logistic regression (LR), linear-support vector machine (SVM), random forest (RF), partial least squares (PLS), ridge, least absolute shrinkage and selection operator (LASSO) and elastic-net regression) were trained using these features. A receiver operating characteristic curve (ROC) analysis was conducted to evaluate the performance of the model and the area under the ROC curve (AUC) was recorded for validation images. Hyper-parameter optimization was performed via grid-search with 20 repeats of ten-fold cross-validation using the caret package^51^ in R. Hyper-parameters of the final selected models are listed in the Supplementary Material.

The same features selected from the heterogenous dataset were then selected from the homogenous dataset and validation set AUC of the classifiers recorded. AUC values between the heterogenous and homogenous datasets were compared using bootstrap with the pROC package in R^52^, and p-values were recorded.

**Figure 4:**
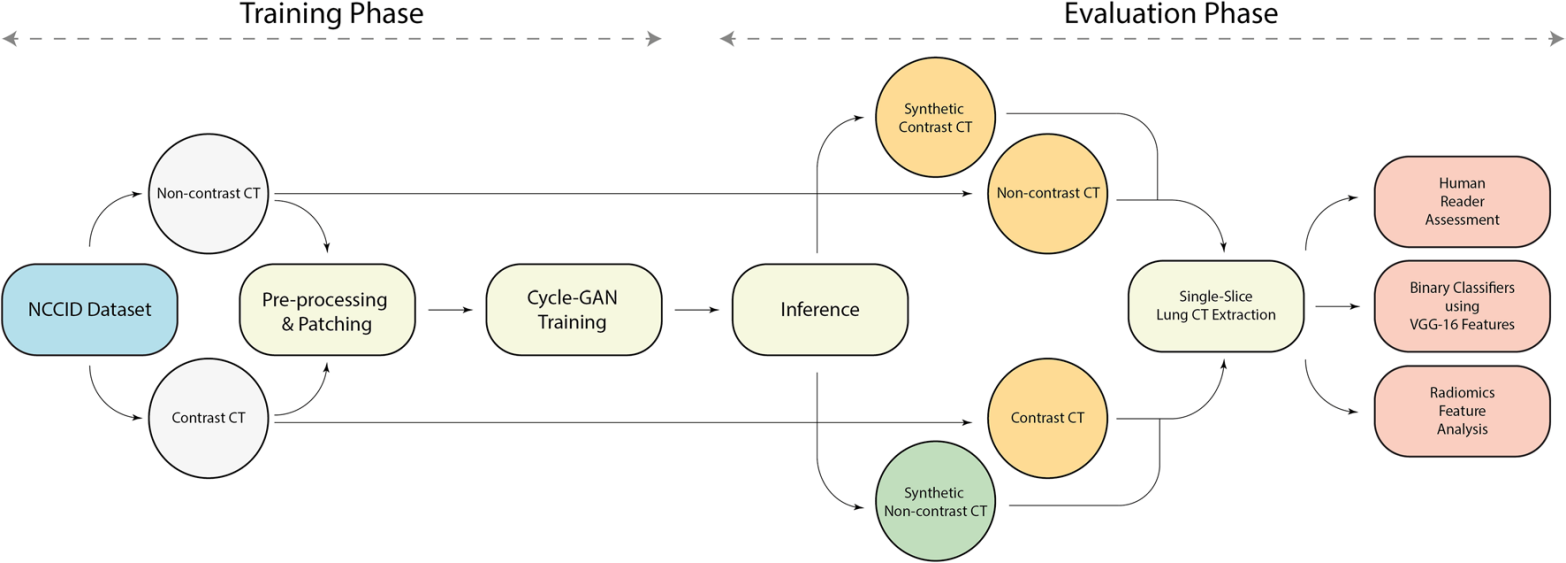
Overview of the proposed COVID-19 data generalization framework. In the training phase, random patches of contrast and non-contrast CT images are selected from pre-processed image volumes and passed to the cycle-GAN network. In the evaluation phase, the synthetic images are generated from the trained weights using sliding window inference. Finally, (**a**) the chest CT slices are qualitatively evaluated in an expert reader assessment, (**b**) the VGG16 features from predicted images are extracted and used in a binary classifier to evaluate our synthesis framework, and (c) radiomics features are compared between acquired and synthetic cycle-GAN-generated images.

For completeness, this experiment was repeated in reverse, first selecting features from the homogenous dataset, and then comparing performance of the classifiers with these same features selected from the heterogenous dataset (results detailed in the supplementary material). The schematic of our proposed framework is shown in Fig. 4.

### Patient consent

Patient consent was not required for this study. Please see the section on Datasets & Ethical Approval for further information.

## Results

**Figure 5:**
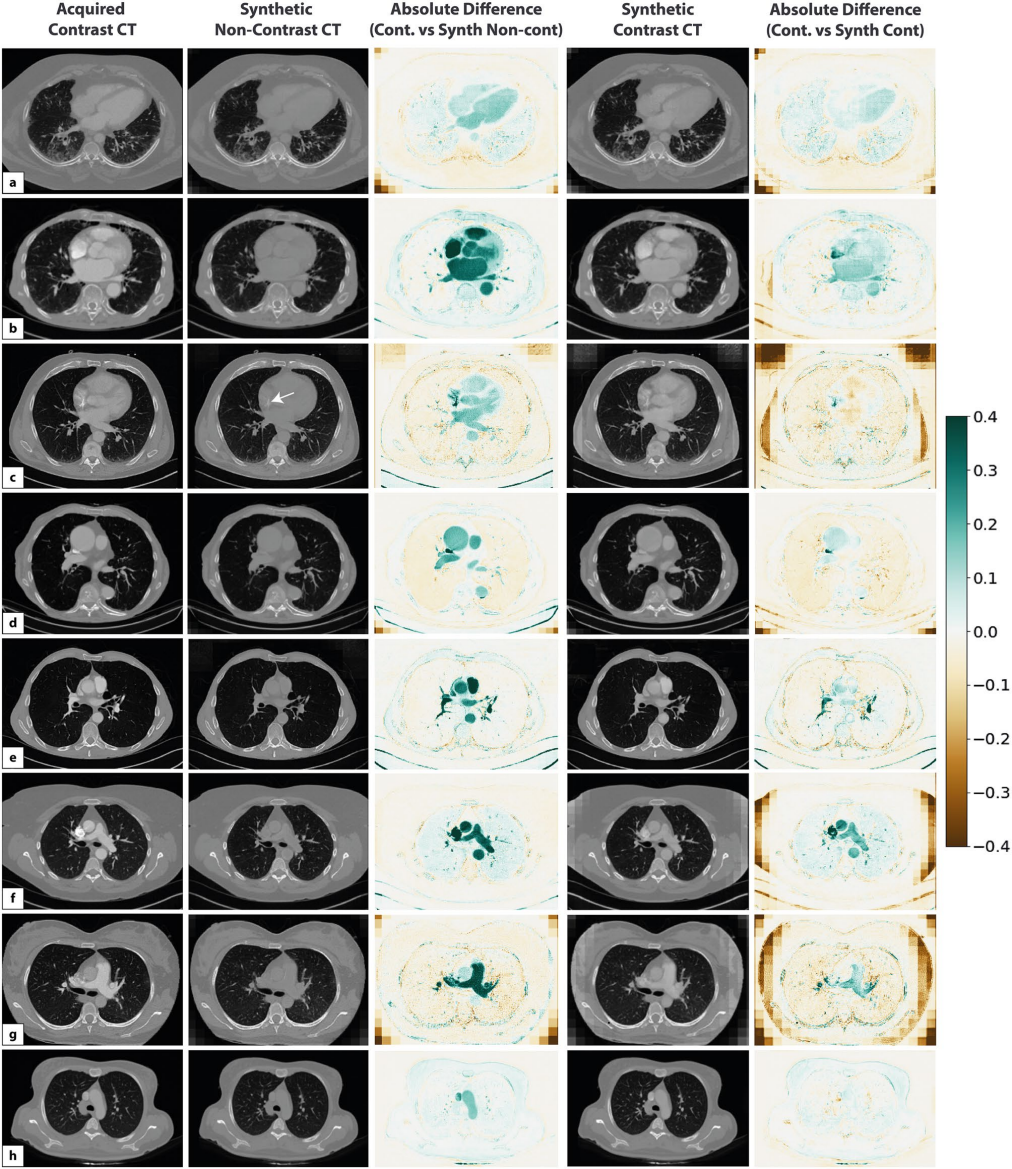
Results of cycle-GAN training on eight example test cases (**a**–**h**) for contrast to non-contrast CT synthesis. The absolute intensity difference maps show the scaled intensity differences between normalized acquired contrast/synthetic non-contrast and synthetic contrast/acquired contrast CTs (intensity range: −400, 400HU; normalized intensity range: −4.0, 4.0). The framework successfully removed and predicted contrast on test chest CTs (e.g. **a**,**d**,**h**). Contrast regions within synthetic contrast images were mainly removed from pulmonary arteries, with some cases showing intensity differences due to variable contrast agent injection timepoints (e.g. **b**,**e**,**f**,**g**). The white arrow represents structures where hypo-intensity remains on chest CTs after synthesis (e.g. **c**).

The examples of the inferred synthetic non-contrast and synthetic contrast CT images predicted from synthetic non-contrast CT are shown in Fig. 5. The absolute intensity difference maps reveal that the trained generators in our framework were able to successfully learn the correct anatomy for contrast removal/transfer despite the heterogeneity in the NCCID dataset. However, in cases with pulmonary angiograms, contrast from hyperdense regions within the chest CT were not fully removed (e.g. Figure 5c). On the other hand, our results demonstrated that the proposed cycle-GAN framework produced photorealistic synthetic contrast CTs from the test dataset, with high visual fidelity closely representing the sharpness and contrast of acquired images. While some differences in contrast intensities were observed on the intensity difference maps (e.g. Fig. 5b–f), the correct structures were identified by the network with no obvious changes to other anatomies on patient scans.

### Expert human reader assessment

**Figure 6:**
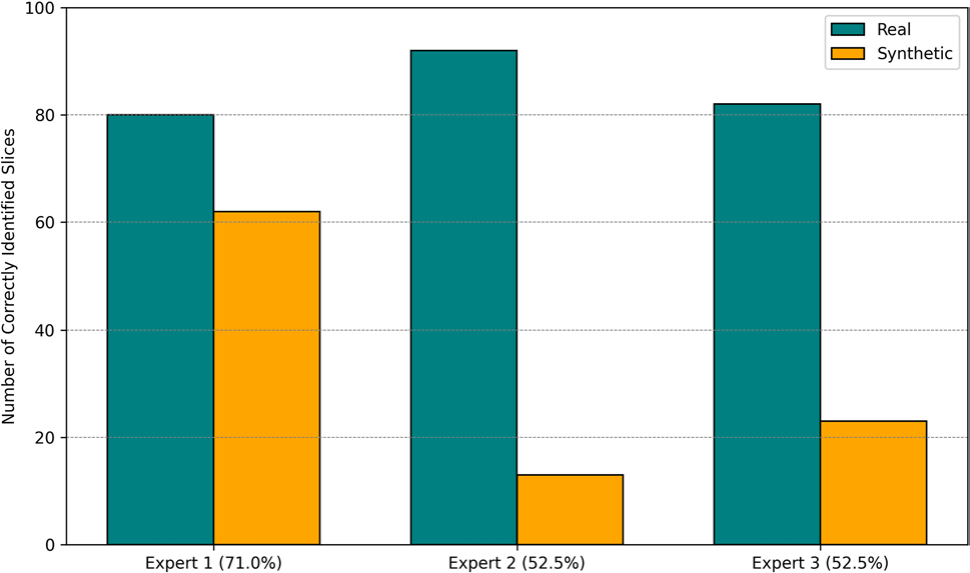
The modified Turing-test of 3 expert readers for blindly classifying 200 chest CT slices (100 synthetic, 100 real). For all readers the number of synthetic images identified wrongly as real CTs were greater than real images classified as synthetic.

The results from our human reader assessment revealed that the experts achieved a mean accuracy of 58.7%, with individual classification accuracies of 62%, 13% and 23% for synthetic CT images (Fig. 6). Sensitivity, specificity and AUC for each reader is shown in Table 1. The false positive rate, indicating the instances where the experts failed to correctly identify the synthetic CT images was 67%. The Fleiss’ Kappa metric was 0.06 (z-score 1.44, p-value 0.15), demonstrating only very slight agreement among the human expert readers^53^.

**Table 1:** Sensitivity, specificity, and AUC values for the human reader assessment.

Reader	Sensitivity	Specificity	AUC
Expert 1	0.8	0.62	0.71
Expert 2	0.92	0.13	0.525
Expert 3	0.82	0.23	0.525

### Validation with a VGG16 feature classifier

**Figure 7:**
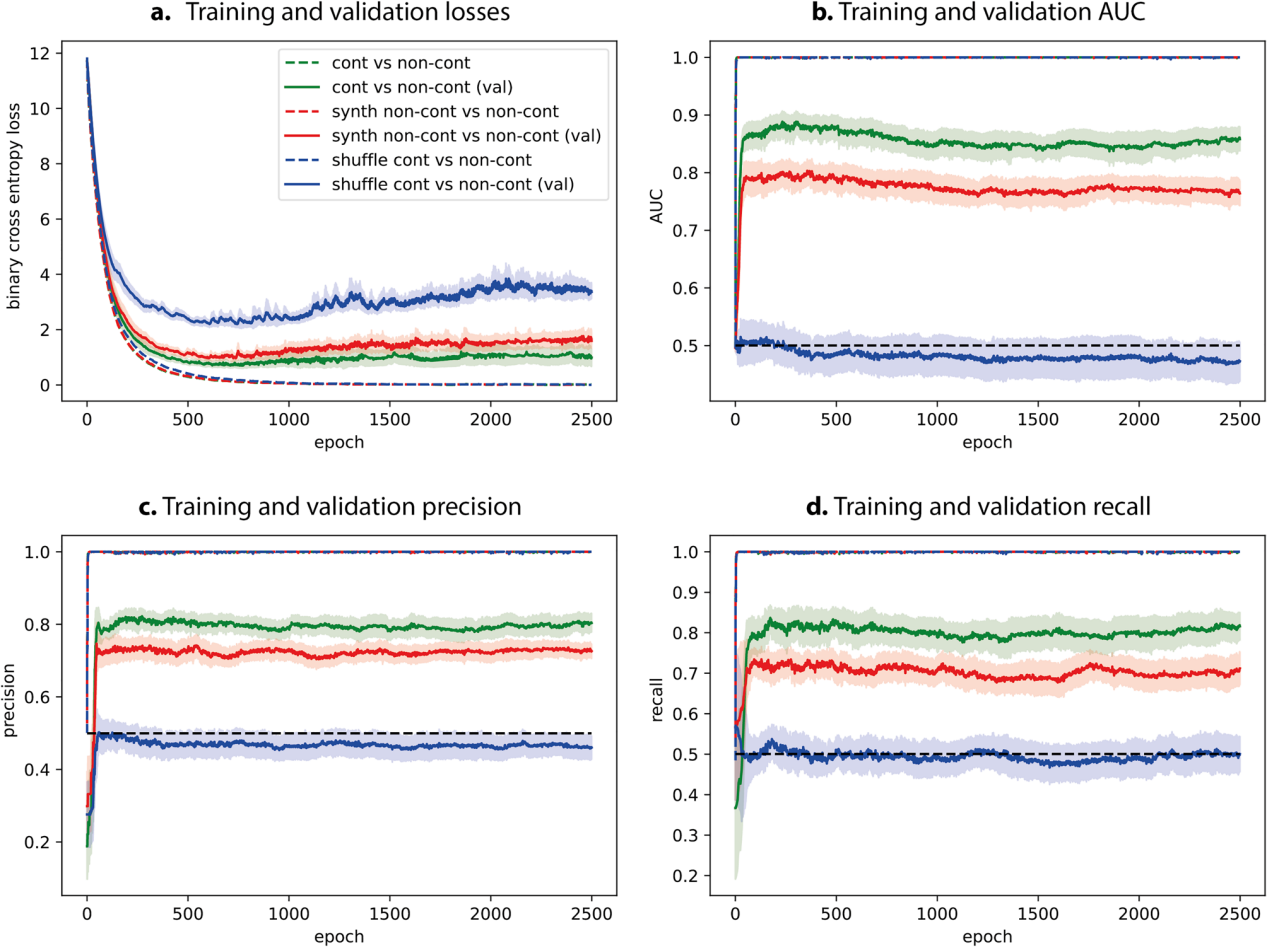
Training and validation curves of the binary classifier applied to acquired contrast/acquired non-contrast, synthetic non-contrast/acquired non-contrast and label shuffled acquired contrast/acquired non-contrast images. Training and validation (**a**) loss, (**b**) AUC, (**c**) precision and (**d**) recall metrics from all classifiers. For each metric the mean values are shown alongside the error margins (shown in pastel colors) from cross validation training. The legend for (**a**) also applies to (**b**–**d**).

Demonstrated in Fig. 7 are the training (dotted) and validation (solid) curves from our VGG16 classifiers for discriminating (i) acquired contrast from acquired non-contrast scans (green) and (ii) synthetic non-contrast from acquired non-contrast scans (red). For all explored metrics, the performance of classifier (ii) demonstrates inferior performance than classifier (i). This indicates that the synthetic non-contrast scans generated by our cycle-GAN are able to fool the classifier, further supporting the evidence found in the reader study. However, classifier (ii) still demonstrates a degree of predictive power that is significantly better than random choice (blue curve), suggesting that there exist subtle features within the synthetic non-contrast scans that are not visible to human readers but can be extracted by a VGG16 classifier.

### Validation with a handcrafted radiomics classifier

Contrast-heterogenous and synthetically homogenized validation set AUC values for 7 classifiers (trained using features selected from the heterogenous images) are detailed in Table 2. LASSO and Elastic Net were the highest performing classifiers on the heterogenous validation set. When models were applied to the contrast-homogenised data, absolute AUC values decreased for all classifiers, with wider confidence intervals (CI), however this was not found to be statistically significant at the 5% level except for the SVM classifier.

**Table 2:** Validation set AUC values and confidence intervals (CI) for 7 machine learning classifiers based on the original contrast-heterogenous dataset and GAN synthesized contrast-homogenous data. P-values were calculated by comparing corresponding classifier ROC curves using bootstrapping.

Classifier	Heterogenous data Validation set		Homogenous data Validation set		P-value
	AUC value	CI	AUC value	CI	
LR	0.9	0.80–1	0.77	0.62–0.92	0.095
SVM	0.9	0.80–1	0.75	0.62–0.88	0.015
RF	0.84	0.72–0.96	0.76	0.61–0.92	0.215
PLS	0.91	0.81–1	0.8	0.66–0.94	0.146
Ridge	0.87	0.75–0.98	0.81	0.68–0.94	0.317
Lasso	0.92	0.83–1	0.82	0.69–0.95	0.138
Elastic Net	0.92	0.83–1	0.80	0.67–0.94	0.102

**Figure 8:**
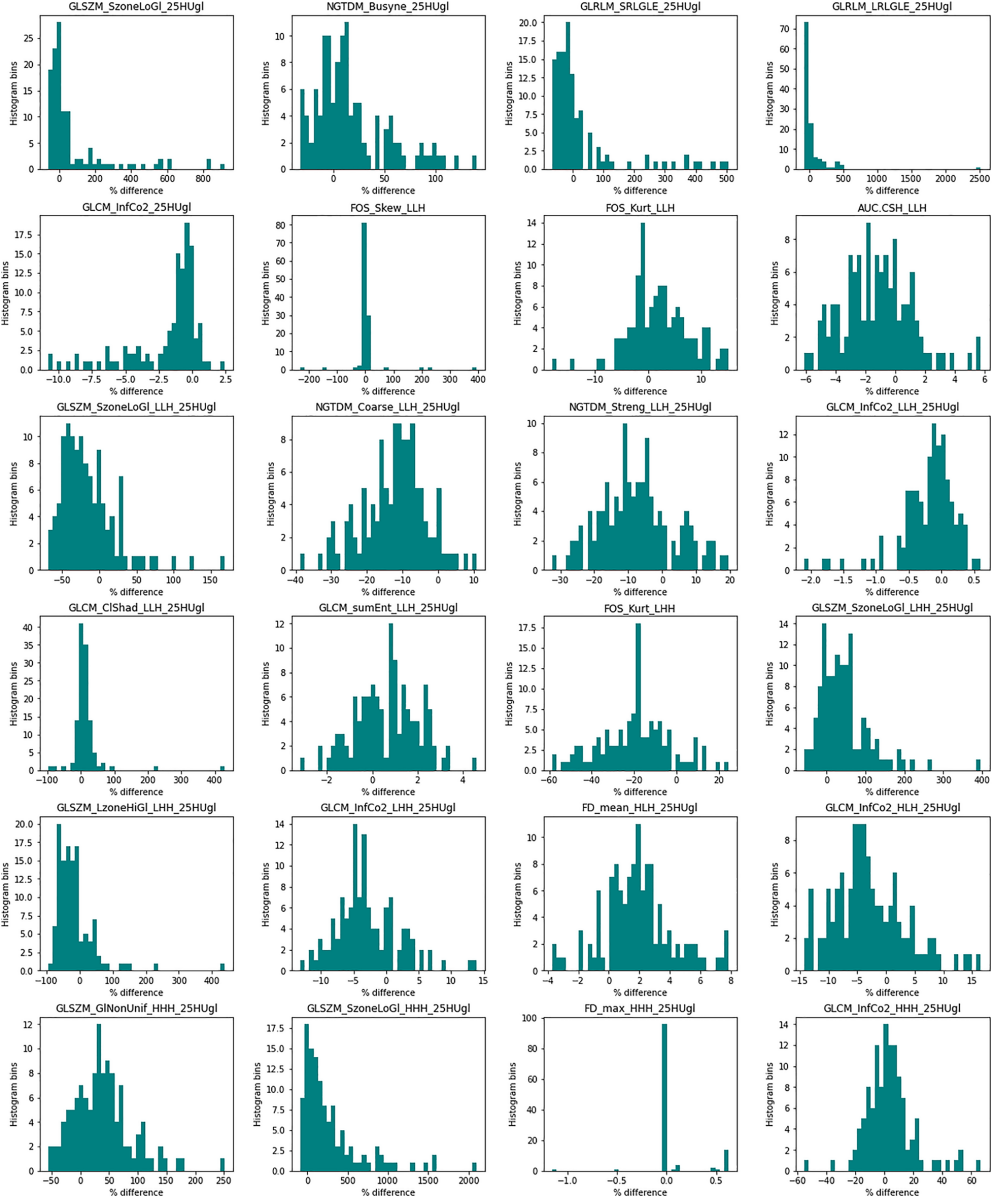
Histograms of the absolute percentage difference in the values of the 24 radiomic features that were used for modeling before and after non-contrast images had the GAN applied. X-axis values pertain to percentages. There is a marked percentage difference, suggesting that the cycle-GAN is augmenting images and subsequently the radiomic features extracted from them.

We further explored the impact of the cycle-GAN on the raw handcrafted radiomic feature values. Figure 8 shows histograms of the absolute percentage difference in the raw radiomic features derived from only the non-contrast images, before and after the cycle-GAN inference was applied. We noted a marked percentage difference of feature values after the GAN was applied, for example up to 500% change for GLRLM_LRLGLE_25HUgl, again indicating that the cycle-GAN model caused visually imperceptive differences in the output images that are nonetheless important in the context of radiomics-based studies.

## Discussion

In response to the global need for improved detection and diagnosis of COVID-19 pneumonia, research attention has been directed towards developing generalizable and efficient AI algorithms that can be applied to large and multi-centre datasets. In this study, we developed a 3D patch-based cycle-GAN to synthesize non-contrast images from contrast-enhanced CTs, using a multi-centre dataset containing 2078 CT scans from 1,650 patients with COVID-19. We hypothesized that this approach could provide contrast-homogenized datasets which may improve performance of future imaging-based AI models. Whilst our investigation primarily focuses on evaluating the efficacy of this technique in the context of COVID-19, its clinical utility may be even wider-reaching, for example finding applications in cancer care. Contrast-enhanced CT plays a key role in lesion detection, characterization, and staging, as well as for radiotherapy treatment planning, guidance, and response assessment^54–56^. Whilst non-contrast CT is less commonly used in these settings, it does provide utility in differential diagnosis, for example in depicting hemorrhage or calcification and by serving as reference images to evaluate the degree of enhancement on contrast-enhanced CT^56^. Contrast-enhanced CT is also widely used in planning radiotherapy to better delineate target volumes and OARs. However, it has been suggested that this may lead to dosimetric errors because of overestimations in tissue electron density^56^. It would therefore be advantageous to automatically synthesize non-contrast images from contrast CT scans, avoiding the need for additional acquisition of non-contrast CTs. Such approaches would be advantageous to reduce demands on resource-constrained health systems.

Cycle-GAN is a promising technique in medical imaging research because of its ability to generate photorealistic images from unpaired data^31,35,37–41^. However, few previous studies have conducted an in-depth analysis of this technique in the context of handcrafted radiomics or qualitative reader assessments. Earlier studies have reported the use of CNNs^34^, cycle-GAN^33,35^ and RadiomicGAN^57^ for CT standardization, however these techniques rely on paired input images for training which limits their applicability in most real-world scenarios. Selim et al. proposed a framework inspired by cycle-GAN that successfully homogenized CT scans from different vendors using their 2D CT harmonization model (CVH-CT)^58^. Although they validated their results based on radiomic features, clinical evaluation of their results was not explored.

In this study, we employed VGG16-based DL and handcrafted radiomics classifiers, along with human reader assessments, to evaluate the technical and clinical aspects of GAN-generated images using 3D patch-based training. Our human reader assessment, which involved two radiologists and one clinical oncologist, revealed that the experts failed to correctly identify the synthetic CT images in 67% of cases (false positive rate). Figure 6 shows that, particularly for Experts 2 and 3, most of the synthetic images were labelled as “real” by the human readers. The mean AUC for the readers was 0.59 and the interrater reliability measurement showed only very slight agreement among the readers, indicating that the readers’ judgement on images were different on cases across our test cohort. These results suggest that the human readers were unable to distinguish consistently and accurately synthetic non-contrast from acquired non-contrast images.

We employed a VGG16 to assess the effectiveness of a DL model in distinguishing between acquired and synthetic non-contrast CT images. Even though there was a decrease in classification accuracy when using homogenized datasets, the classifier was still able to differentiate between the two groups better than chance, supporting the notion that there exist nuanced features within the synthetic non-contrast scans that can influence the VGG16 classifier's performance. Whilst there was a slight increase in validation binary cross entropy loss, suggesting some overfitting, the validation curves plateaued for AUC, precision, and recall, indicating that the training was stable.

Contrast is known to impact upon the performance of radiomic models^22,59^. In our experiment using a handcrafted radiomics classifier, we hypothesized that replacement of contrast images with GAN-generated synthetic non-contrast images would improve performance of a radiomic classifier trained to distinguish COVID-19 vs non-COVID-19 pneumonia. We were unable to reject the null hypothesis in this experiment, likely due to the GAN radiomic features being sensitive to subtleties within the synthetic images that were not appreciable by eye, further validating our VGG16 classification experiment. Our findings suggest that the use of synthetically homogenized datasets may impair the discriminatory ability of classifiers based on radiomic features. Histograms of absolute percentage difference in the features from non-contrast scans before and after synthesis support that, whilst cycle-GAN produced photorealistic images that were sufficient to pass a modified Turing test, they were not identical at the radiomic feature level.

Overall, our results demonstrate that unsupervised DL algorithms can effectively learn global contexts from large datasets and generate realistic predictions sufficient to pass human reader assessments. However, the synthetic images are distinguishable from acquired images by VGG16-DL and handcrafted radiomics classifiers, likely due to underlying subtleties introduced by the cycle-GAN.

One of the challenges of synthesizing non-contrast images from contrast CT is the difference in image acquisition parameters, which can result in variations in texture and contrast distribution^14,15,60^. This was evident in absolute intensity difference maps of some cases that displayed slight variations in texture for synthetic images. Additionally, the heterogeneity in contrast distribution amongst patients may affect the success of cycle-GAN. The NCCID dataset included CT pulmonary angiograms that had marked differences in contrast distribution at the time of acquisition compared to CTs performed for evaluation of lung parenchyma. In these cases, unrealistic non-contrast CT synthesis occurred for some hypo-intense structures. Another consideration is that GANs are notoriously difficult to train and our model may have converged to local minima^61^. Despite these challenges, our framework was able to generate plausible synthetic contrast chest CTs from test patients.

A limitation of this study was the use of small patches for training the cycle-GAN framework. Whilst patch-based methods provide more training examples, they also lead to a reduction in field-of-view (FOV) of training images. We overcame this limitation by using stochastic training and dense inference to generate the final synthetic volumes. However, further research is needed to compare the predictive performance of models with varying input sizes. The use of single-slice ROIs for feature extraction in the radiomics experiment and the variability in contrast distribution due to the timing of contrast injection (including angiograms and portal venous phase images) were additional limitations of our experiments. Our radiomics analysis was limited by the small dataset, although we were still able to obtain statistically significant results for the SVM classifier. In addition, the non-COVID-19 dataset had an imbalance of contrast and non-contrast images, unlike the balanced dataset of COVID-19 patient scans. However, our AUC values were higher than would be expected if the classifiers were classifying purely based on contrast-status alone.

Further work with a larger radiomics dataset and multi-slice volumes is warranted.

## Future work

Though further investigation is required to evaluate the diagnostic abilities of synthetic non-contrast CTs for automated predictions, our experiments provide valuable insight into the use of unsupervised synthesis techniques, such as cycle-GAN, as data augmentation or homogenization strategies. Future studies on unsupervised style-transfer should incorporate additional metrics that guide GANs to not only produce realistic predictions but closely represent the underlying information (e.g. radiomic features) from training images. Furthermore, training DL networks using large and multi-centre data can be influenced by bias, due to factors such as contrast/non-contrast data distribution, image acquisition parameters, or scanner type, which may hinder the cycle-GAN’s performance in effectively learning the optimal mapping between both sets of input images. Utilizing more controlled training datasets or introducing intermediate scan homogenization techniques may further assist the framework learning process, however, caution must be exercised in use of chained-AI algorithms to avoid error propagation and diminishing returns.

## Conclusion

Handcrafted and DL CT-based radiomic models are increasingly being used both for COVID-19 and other health conditions in the context of COVID-19 endemicity^1–5^. Such models require large datasets, however, may suffer from contrast/scan heterogeneity, adversely affecting model generalizability. We developed and evaluated a cycle-GAN model using a multi-centre dataset of COVID-19 pneumonia for data homogenization. Though synthetic non-contrast images generated by our cycle-GAN model passed human assessment, our findings indicate the presence of subtle features in the synthetic images that are detectable at the radiomic feature level. This implies caution is warranted before GAN-synthesized images are used for data homogenization prior to handcrafted or DL radiomic modeling. Whilst our model shows promise in addressing contrast heterogeneity, it also highlights challenges associated with large-scale datasets and the need for more controlled training datasets or intermediate homogenization steps for improved performance. Overall, our study underscores the need for further research to optimize the use of GANs for medical imaging and improve their clinical utility.

## Summary

Handcrafted and deep learning (DL) radiomics are two common imaging-based artificial intelligence (AI) techniques that have been leveraged to develop computed tomography (CT) imaging-based models for COVID-19 and other healthcare research. Such approaches require large datasets for training and analysis, however contrast heterogeneity from real-world medical imaging datasets may impair model performance.

In this study, using a multi-centre dataset containing 2078 CT scans from 1,650 patients with COVID-19, we developed a 3D patch-based cycle-GAN to synthesize non-contrast images from contrast-enhanced images to homogenize CT data for the development of future COVID-19 AI models. We initially hypothesized that as COVID-19 reaches endemicity, our framework may offer superior applicability to future CT imaging datasets, compared to those developed in the pre-COVID era.

We evaluated the performance of our contrast to non-contrast cycle-GAN to assess network generalizability as a contrast homogenization tool with both a handcrafted radiomic and VGG DL classification task. In a modified Turing-test, human experts identified synthetic vs original images, with an average score of 58.7% (range 52.5–71%) attesting to the photorealism of the synthetic images. Furthermore, Fleiss’ Kappa was 0.06 (p-value 0.15), demonstrating only slight agreement among human experts. However, on testing performance of machine learning classifiers with radiomic features, performance decreased with use of synthetic images. Marked percentage difference was noted in feature values between pre- and post-GAN non-contrast images. Our results suggested that our cycle-GAN model produced synthetic non-contrast images sufficient to pass human assessment, subtle features existed in the synthetic images, potentially introduced by the cycle-GAN or inference strategy which are detectable at the radiomic feature level. This implies caution is warranted before GAN-synthesized images are used for data synthesis prior to radiomic modeling or further clinical studies.

## Supplementary Information

Supplementary Information.

## Data Availability

Due to confidentiality, data collected for the study are not publicly available for download, however the corresponding authors can be contacted for academic inquiries. Tools for deep learning are indicated in the methods section.
