# Supplementary material for: Non-contrast CT synthesis using patch-based cycle-consistent generative adversarial network (Cycle-GAN) for radiomics and deep learning in the era of COVID-19
Source: Sci Rep. 2023 Jun 29;13:10568. doi: 10.1038/s41598-023-36712-1 (PMC10310777; doi:10.1038/s41598-023-36712-1)
Supplement: Supplementary file 1 — Supplementary Information. [file 41598_2023_36712_MOESM1_ESM.docx]

**Supplementary Material –** **Non-Contrast CT Synthesis using Patch-Based Cycle-Consistent Generative Adversarial Network (Cycle-GAN) for Radiomics and Deep Learning in the Era of COVID-19**

Sumeet Hindocha*^1,2,4,5^, Reza Kalantar*^1^, Benjamin Hunter^4,5^, Bhupinder Sharma^1,3^, Nasir Khan^3^, Dow-Mu Koh^3^, Merina Ahmed^6^, Eric O. Aboagye^4^, Richard W. Lee†^5^, Matthew Blackledge†^1^

*=joint first authors, †=joint supervising authors

Affiliations:

^1^Division of Radiotherapy and Imaging, the Institute of Cancer, London, SM2 5NG, United Kingdom

^2^AI for Healthcare Centre for Doctoral Training, Imperial College London, Exhibition Road, London, SW7 2BX, United Kingdom

^3^Department of Radiology, The Royal Marsden NHS Foundation Trust, Sutton, SM2 5PT, United Kingdom

^4^Cancer Imaging Centre, Department of Surgery & Cancer, Imperial College London, Du Cane Road, London, W12 0NN, United Kingdom

^5^Early Diagnosis and Detection Team, The Royal Marsden NHS Foundation Trust, Fulham Road, London, SW3 6JJ, United Kingdom

^6^Lung Unit, The Royal Marsden NHS Foundation Trust, Sutton, SM2 5PT, United Kingdom

**Table of Contents**

Further details on the handcrafted radiomic classifier experiment:

- Details on the non-COVID-19 pneumonia dataset
- List of 24 features remaining after Kendall’s rank feature selection
- Summary of the seven machine learning classifiers used
- Supplementary Table 1: Hyper-parameters for the seven machine learning classifiers used
- Results of the experiment in reverse

**The non-COVID-19 pneumonia dataset:**

This dataset contained thoracic CT images of patients from 3 UK National Health Service (NHS) Trusts: The Royal Marsden NHS Foundation Trust, Imperial College Healthcare NHS Trust and Manchester University NHS Foundation Trust. Eligible patients were those:

- With new radiological lung changes on CT (confirmed on report) of a severity and distribution consistent with lower respiratory tract infection
- And laboratory findings that fulfil one or more of the following criteria of infection: Nasopharyngeal aspirate or swab positive for a respiratory virus by PCR; Sputum sample or bronchial washings positive MCS for an organism(s) consistent with lower respiratory tract infection, cytology or beta-glucan/galactomannan positive for PCP or fungal infection, positive urine legionella/pneumococcal antigen screen, positive serology for mycoplasma pneumonia; broncho-alveolar lavage for markers of infection (MCS, PCR, fungal culture, beta-glucan/galactomannan for PCP or other evidence of lower respiratory tract infection (including invasive fungal infection) by cytology. Where no such laboratory findings were positive but the patient improved with anti-microbial therapy, such cases were eligible at the discretion of a respiratory physician and radiologist with lung interest, after review of the case-notes and imaging.
- Not previously treated with immunotherapy
- Who did not have radiotherapy involving the thorax in the 12 months prior to presentation
- First assessed prior to 1st January 2020 (and therefore not attributable to COVID-19)

**Features remaining after Kendall’s rank feature reduction:**

Kendall’s rank correlation is a “filter” feature selection method which relies only on the characteristics of features independently of any machine learning model. It is non-parametric and assumes a monotonic relationship between variables. It is preferred where dataset have a limited number of observations or contain outliers. For our study we used the corr package and specified that the top 20% (24) features be included in the feature set following Kendall’s rank correlation. These 24 features were:

1. GLSZM_SzoneLoGl_25HUgl
2. NGTDM_Busyne_25HUgl
3. GLRLM_SRLGLE_25HUgl
4. GLRLM_LRLGLE_25HUgl
5. GLCM_InfCo2_25HUgl
6. FOS_Skew_LLH
7. FOS_Kurt_LLH
8. AUC.CSH_LLH
9. GLSZM_SzoneLoGl_LLH_25HUgl
10. NGTDM_Coarse_LLH_25HUgl
11. NGTDM_Streng_LLH_25HUgl
12. GLCM_InfCo2_LLH_25HUgl
13. GLCM_ClShad_LLH_25HUgl
14. GLCM_sumEnt_LLH_25HUgl
15. FOS_Kurt_LHH
16. GLSZM_SzoneLoGl_LHH_25HUgl
17. GLSZM_LzoneHiGl_LHH_25HUgl
18. GLCM_InfCo2_LHH_25HUgl
19. FD_mean_HLH_25HUgl
20. GLCM_InfCo2_HLH_25HUgl
21. GLSZM_GlNonUnif_HHH_25HUgl
22. GLSZM_SzoneLoGl_HHH_25HUgl
23. FD_max_HHH_25HUgl
24. GLCM_InfCo2_HHH_25HUgl

**Summary of machine learning algorithms:**

The models used in this study are supervised classification algorithms. A summary of each is provided below.

LR – generalised linear model is a generalisation of linear regression to modelling dependencies between predictors and dependent features. Logistic regression is a form of GLM used in this study. It uses the logistic sigmoid function to return a probability value which can then be mapped to two or more separate classes^1^.

L-SVM – linear support vector machines plot training samples and assigns a hyperplane (decision boundary) to separate these into classes. The optimal hyperplane is that which maximises the distance between data-points^2^.

RF – Random Forest is an ensemble decision-tree based model. RF uses bagging and feature variability when building each decision tree to create an uncorrelated forest whose overall prediction is more accurate than each individual tree^3^.

PLS – Partial Least Squares is a multivariate linear regression model which forms linear combinations of features in a supervised manner. It is able to handle datasets with large numbers of features, high collinearity between features and small numbers of observations^4^.

LASSO and Elastic Net regression can also be used for classification. Ridge is another form of regression where the loss function is modified to minimize the complexity of the model. Here alpha = 0.

**Supplementary Table 1:** Hyperparameters used for the Radiomic models.

| **Algorithm** | **Library** | **Hyperparameter** |
| --- | --- | --- |
| LR | glm | - |
| SVM | *svmLinear* | C = 1 |
| RF | *rf* | mtry = 5 |
| PLS | *pls* | ncomp = 5 |
| Ridge | *glmnet* | alpha = 0, lambda = 0·1519911 |
| Lasso | *glmnet* | alpha = 1, lambda = 0·01232847 |
| Elastic Net | *glmnet* | alpha = 0.5, lambda = 0·03222285 |

**Results of the experiment in reverse:**

Here features were first selected from the homogenous dataset, and then performance of the classifiers compared with these same features selected from the heterogenous dataset). The 24 features selected were:

1. FOS_Imode
2. GLSZM_SzoneLoGl_25HUgl
3. FD_max_25HUgl
4. FD_max_LLH_25HUgl
5. GLCM_ClShad_LLH_25HUgl
6. FOS_Imin_LHH
7. FOS_Kurt_LHH
8. GLSZM_SzoneLoGl_LHH_25HUgl
9. GLSZM_LzoneHiGl_LHH_25HUgl
10. NGTDM_Busyne_LHH_25HUgl
11. NGTDM_Streng_LHH_25HUgl
12. FD_min_LHH_25HUgl
13. GLCM_AutoCorrel_LHH_25HUgl
14. FOS_Skew_HLH
15. FOS_Kurt_HLH
16. FD_lacunarity_HLH_25HUgl
17. FD_max_HLH_25HUgl
18. GLCM_ClShad_HLH_25HUgl
19. FOS_Imin_HHH
20. GLRLM_LRLGLE_HHH_25HUgl
21. FD_max_HHH_25HUgl
22. FD_min_HHH_25HUgl
23. GLCM_InfCo2_HHH_25HUgl
24. GLCM_AutoCorrel_HHH_25HUgl

Validation set AUC values for the 7 machine learning classifiers were:

| Classifier | Homogenous data validation set | Heterogenous data validation set | P-value |
| --- | --- | --- | --- |
| LR | 0.77 | 0.85 | 0.277 |
| SVM | 0.7 | 0.75 | 0.495 |
| RF | 0.68 | 0.71 | 0.702 |
| PLS | 0.76 | 0.86 | 0.224 |
| Ridge | 0.78 | 0.76 | 0.639 |
| Lasso | 0.78 | 0.84 | 0.494 |
| Elastic Net | 0.79 | 0.84 | 0.490 |

**Supplementary Material References:**

1 Venables WN, Ripley BD. Generalized Linear Models. In: Modern Applied Statistics with S. New York, NY: Springer New York, 2002: 183–210.

2 Karatzoglou A, Hornik K, Smola A, Zeileis A. kernlab - An S4 package for kernel methods in R. *J Stat Softw* 2004; **11**: 1–20.

3 Breiman L. Random forests. *Mach Learn* 2001; **45**: 5–32.

4 Mevik B-H, Wehrens R. Introduction to the pls Package. Help Sect. ‘pls’ Packag. RStudio Softw. 2015; : 1–23.
